# Too late, too often: missed opportunities in male bone health: a real-world portrait from a 14-year specialist referral experience

**DOI:** 10.1007/s40618-025-02753-8

**Published:** 2025-12-29

**Authors:** Sara De Vincentis, Antonino Russo, Erica Taliani, Anna Ansaloni, Daniela Domenici, Giulia D’Angelo, Veronica Demichelis, Bruno Madeo, Vincenzo Rochira

**Affiliations:** 1 Endocrinology, Department of Biomedical, Metabolic and Neural Sciences, University of Modena and Reggio Emilia, Modena, Italy; 2 Unit of Endocrinology, Department of Medical Specialties, Azienda Ospedaliero-Universitaria Policlinico di Modena, Ospedale Civile di Baggiovara, Via Giardini, 1355, Modena, Italy

**Keywords:** Male osteoporosis, Fractures, Secondary osteoporosis, Gender gap, Undermanagement, Hypogonadism

## Abstract

**Purpose:**

To characterize, using real-life data, the clinical profile of men undergoing their first bone health evaluation at a tertiary academic center over a 14-year period.

**Methods:**

Retrospective, observational, cross-sectional study including adult men referred to our center between 2007 and 2021 for bone health assessment. Fractures, comorbidities, risk factors for bone loss, and pharmacological treatments were collected.

**Results:**

536 men were enrolled (147 under 50, 385 over 50). At least one comorbidity associated with bone loss was found in 49.3% of patients, and 43.8% were receiving medications causing bone mineral density (BMD) reduction—mainly corticosteroids and androgen deprivation therapy. The prevalence of osteoporosis, osteopenia, and low BMD for age was 42.3%, 44.8%, and 48.6%, respectively. Osteoporosis-related fractures were found in 216 patients (40.8%), whose 34 men under 50 (15.7%). Up to 17.5% of men with fractures had normal BMD. A total of 181 patients (33.8%) had never received calcium/vitamin D supplementation or bone-active therapy; the prevalence of treatment-naïve patients was 20–23% even among men with fractures or receiving corticosteroids/androgen-deprivation therapy.

**Conclusions:**

Male osteoporosis presents with a high rate of fractures in the real-life clinical practice at a tertiary academic center. The high prevalence of comorbidities associated with bone loss suggests that secondary forms of osteoporosis should be carefully investigated, even in presence of normal BMD. The significant proportion of untreated men—including those with known risk factors or fractures—highlights the urgent need to raise awareness and improve the management of male osteoporosis, especially in primary healthcare.

**Supplementary Information:**

The online version contains supplementary material available at 10.1007/s40618-025-02753-8.

## Introduction

Osteoporosis represents a significant social and healthcare burden, a trend expected to worsen with the progressive aging of the global population. Although commonly perceived as a condition primarily affecting women, osteoporosis in men has emerged as a relevant health issue, with an estimated lifetime fracture risk ranging from 13% to 30% in males over the age of 50 [1–3]. Notably, this fracture risk has not declined in recent years, despite the expanding availability of bone-active pharmacological agents [4].

Important differences exist between men and women regarding the pathophysiology and clinical presentation of osteoporosis. Unlike postmenopausal women, men do not experience the rapid bone loss associated with a sudden decline in sex steroid levels. Instead, beginning around the sixth decade of life, men undergo a gradual reduction in testosterone levels, which in turn contributes to progressive bone loss [5, 6]. Furthermore, osteoporotic fractures in men are associated with greater morbidity and mortality compared to women [7–10].

Secondary causes of osteoporosis are often underrecognized in both sexes, particularly in men [11]. Secondary osteoporosis accounts for most of the osteoporotic fractures in men (up to 60% in men with fractures) [12, 13], but this esteem is based on limited data [14, 15].

Over the past decade, male osteoporosis has received increasing attention [16], and few clinical guidelines have been developed for its management [17, 18]. Nevertheless, most research and operational criteria remain predominantly focused on women. As a result, osteoporosis in men continues to be underdiagnosed and undertreated, even following an initial fracture event [16, 19]. Given the increased life expectancy of men, the substantial burden associated with fractures in the elderly, and the availability of effective pharmacological treatments, it is critical to improve the identification of men at high risk for osteoporosis-related fractures [20, 21]. Several risk factors for low bone mineral density (BMD) and fractures in men have been identified, including excessive alcohol intake, prolonged corticosteroid exposure (endogenous or exogenous), and hypogonadism [22, 23]. Early identification of these risk factors in real-life clinical practice is essential for optimizing strategies and reducing fracture incidence in the male population. However, studies specifically focused on risk factors in men or describing the characteristics of men referring to health care system for bone evaluation remain limited. In light of this gap, the aim of the present study was to describe the clinical characteristics of men undergoing their first bone health assessment at a tertiary, internationally recognized academic medical referral center for the management of male osteoporosis [18, 24], based on real-life data collected over a 14-year observation period.

## Methods

### Study design and participants

This cohort study documented the real-life clinical management of male outpatients referring for the first time to the Endocrinology Unit of University of Modena and Reggio Emilia for a bone health evaluation. The only inclusion criteria were male sex and age over 18 years; no exclusion criteria were applied.

**Fig. 1:**
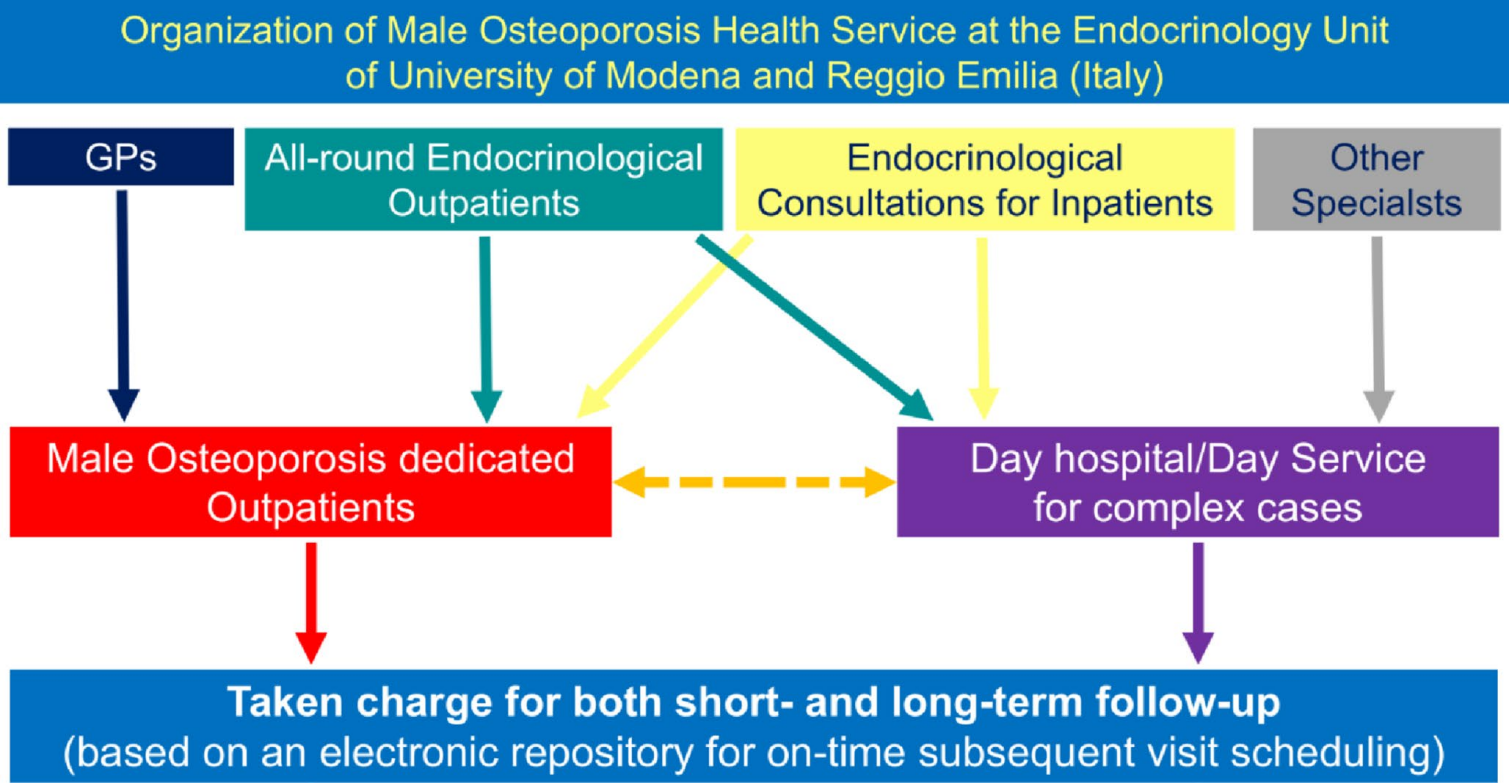
Work-up organization dedicated to male osteoorosis. GP: General Practitioner

Between May 2007 and December 2021, a total of 536 male patients were enrolled. Referrals to the male osteoporosis outpatient clinic were made in accordance with the hospital’s organizational model, which includes:

(i) dedicated appointment slots specifically for male osteoporosis evaluations, accessible to both general practitioners (GPs) and medical specialists; (ii) an integrated electronic referral and follow-up system designed to ensure both short- and long-term continuity of care, while minimizing patient drop-out (Fig. 1). It should be remarked that GPs are actively involved in the management of osteoporosis in terms of diagnosis, prevention, and first line therapy according to the Italian Health Service. This organization is further implemented in the province of Modena where GPs receive periodically continuing medical education on the clinical management of osteoporosis with the aim of leaving in the hands of the GP patients at risk of osteoporosis and less complicated patients with osteoporosis.

## Main outcome measures

We collected from patients’ record charts the following parameters that are routinely explored in the real-life clinical practice at outpatient clinics for osteoporosis: age, weight, height, medication use, prior history of any fracture, lifestyle habits (e.g. smoke, alcohol intake, physical activity, diet calcium intake), other osteoporosis-related risk factors (e.g. familial history of fragility fractures) and medical history to identify any disease/therapy recognized as causes of secondary osteoporosis.

Patients were classified according to the source of referral: (i) endocrinologist; (ii) GP; (iii) other specialists (for example, hematologist or oncologist). This information was obtained from patients’ record charts where the source of referral is recorded.

## Risk factors for osteoporosis and history of fractures

Patient self-reports, clinical history, physical examination (e.g., low body mass index [BMI]), and drug-tracing criteria obtained from patients’ record charts were used to identify the presence or absence of conditions inducing bone loss.

Recorded risk factors for osteoporosis were age >65 years, sedentary lifestyle (muscle-strengthening physical activity and exercise less than 2–3 days/week), low BMI, excessive alcohol intake (daily intake or ≥ 10 units per week or ≥ 3 units/day, current smoking, parental history of fragility fractures, chronic glucocorticoid therapy (≥ 5 mg prednisolone daily or equivalent for 3 months or more), low calcium intake (less than 1000 mg/day evaluated through a dietary interview) [18, 25].

Medical conditions associated with osteoporosis were also explored and into major comorbidities (e.g. primary hyperparathyroidism, hypogonadism, malabsorption/malnutrition, hypercalciuria, type 2 diabetes mellitus, mastocytosis, iron overload disease including thalassemia and hemochromatosis) and minor comorbidities related to bone loss (e.g. hyperprolactinemia, chronic HBV/HCV hepatitis, Parkinson disease, Paget disease) [1, 11, 18]. Besides, the current use of drugs associated with bone loss was investigated, especially glucocorticoids treatment for at least 3 months and/or androgen deprivation therapy [1, 11, 18].

The sum of the number of current risk factors and comorbidities causing bone loss was calculated, as previously described [26].

Furthermore, we considered whether any previous or ongoing bone active therapy treatment (bisphosphonates, denosumab, teriparatide) and/or supplementation with calcium and vitamin D.

Documented prior history of non-traumatic fractures was also ever recorded.

## Bone parameters and calculated risk of fracture

If available, radiological bone parameters assessed by Dual-energy X-ray Absorptiometry (DXA) were recorded.

Consistent with the statements of WHO and ISCD, in men aged >50 years the diagnostic threshold for “osteoporosis” is a BMD T-score level *≤ −* 2.5 SD at almost one site measured, BMD T-score level between − 1 and − 2.5 SD defines “osteopenia”, while BMD T-score > −1 SD defines the “normal BMD range” [17, 18, 27–29]. In men aged < 50 years, BMD Z-score *≤ −* 2 SD, at almost one site measured, is the diagnostic threshold for “bone mass below the expected range for age”, while BMD Z-score > −2 SD defines “bone mass in normal range for age” [17, 18, 27–29].

Patient self-reports and any radiological examinations documenting the presence of vertebral and non-vertebral fractures (e.g. standard x-ray of the spine, vertebral fracture assessment using DXA, magnetic resonance imaging [MRI], or computed tomography) were also collected to evaluate history of fragility fractures.

Fracture risk was calculated through the web-based algorithm of the 10-year Fracture Risk Assessment Tool (FRAX^®^) the adapted for Italy available at http://www.shef.ac.uk/FRAX®. In this way, 2 different scores were obtained for major osteoporotic fracture risk and hip fracture risk. According to the National Osteoporosis Foundation criteria, a FRAX score of ≥ 20% for major fracture or ≥ 3% for hip fracture is defined as a patient at high risk, and these values are considered as the threshold for intervention [30].

### Statistical analysis

Proportions and rates were calculated for categorical data; continuous data were reported as median and interquartile range (IQR).

The non-parametric Mann–Whitney U test was used for comparisons of continuous variables since they resulted not normally distributed at the Kolmogorov–Smirnov test. Categorical variables were compared by Pearson’s Chi-squared test.

Bivariate ordinal logistic regression was used to explore predictive factors for fractures; results of proportional odd models were expressed through odds ratio (OR) and 95% confidence interval. A receiver operator characteristic (ROC) curve was built to define possible cut-off points for the number of risk factors plus comorbidities causing bone loss that better predict the presence of fragility fractures; ROC cut-offs were calculated by the Youden’s index through the identification of the best pair of sensitivity and specificity.

Given the primarily descriptive nature of the analyses, no formal correction for multiple comparisons was applied.Statistical analyses were performed using the Statistical Package for the Social Sciences’ (SPSS) software for Windows (version 28.0; SPSS Inc, Chicago, IL, USA).

For all comparisons, *p* < 0.05 was considered statistically significant.

## Results

A total of 536 men were included in the study: 74 (13.9%) were aged 18–40 years, 73 (13.7%) aged 41–50, 88 (16.5%) aged 51–60, 122 (22.9%) aged 61–70, 128 (24.1%) aged 71–80, and 47 (8.8%) aged > 80. Clinical characteristics of the entire cohort are summarized in Table 1.

**Table 1:** Clinical characteristics of the entire cohort. Continuous data are reported as median (IQR)

	Enrolled patients
*N*	536
**Anthropometric characteristics**	
Age (yrs)	62.3 (47.9–73.0)
*18–40*	74 (13.8%)
*41–50*	73 (13.6%)
*51–60*	88 (16.4%)
*61–70*	122 (22.8%)
*71–80*	128 (23.9%)
*> 80*	47 (8.8%)
*n.a.*	4 (0.7%)
Height (m)	1.73 (1.69–1.78)
Weight (kg)	77 (69–85)
BMI (kg/m^2^)	25.3 (23.2–28.1)
**Source of referral**	
Endocrinologist	197 (36.8%)
General practitioner	229 (42.7%)
Other specialists	110 (20.5%)
**Risk factors**,** comorbidities and therapies associated with osteoporosis**	
Age > 65 years	242 (45.4%)
BMI < 18 kg/m^2^	7 (1.3%)
Familial history of osteoporosis	134 (27.6%)
Parental history of hip fracture	44 (9.3%)
Smoke	166 (31.8%)
Alcohol consumption > 3 units/day	27 (5.3%)
Inconstant physical activity	393 (78.6%)
Low dietary calcium intake	44 (8.8%)
FRAX score for hip fracture (%)	2.3 (0.9–6.2)
FRAX score ≥ 3% for hip fracture	204/471 (43.3%)
FRAX score for major fracture (%)	6.5 (3.6–12.0)
FRAX score ≥ 20% for major fracture	43/469 (9.2%)
Comorbidities < 2	226 (42.2%)
Comorbidities *≥* 2	310 (57.8%)
**Vitamin D status**	
≤ 10 ng/ml	31 (5.8%)
11–20 ng/ml	128 (23.9%)
21–30 ng/ml	198 (36.9%)
> 30 ng/ml	139 (25.9%)
Not available	40 (7.5%)
**Management for bone health before bone specialist’s evaluation**	
Neither prior calcium and/or vitamin D supplementation nor drugs	181 (33.8%)
Prior calcium and/or vitamin D supplementation, no drugs	219 (40.8%)
Ongoing calcium-vitamin D supplementation, prior anti-osteoporotic medications	26 (4.9%)
Ongoing drugs plus calcium and/or vitamin D supplementation	99 (18.5%)
Ongoing drugs without calcium-vitamin D supplementation	11 (2.1%)

n.a.: not available. FRAX: 10-year Fracture Risk Assessment

**Fig. 2:**
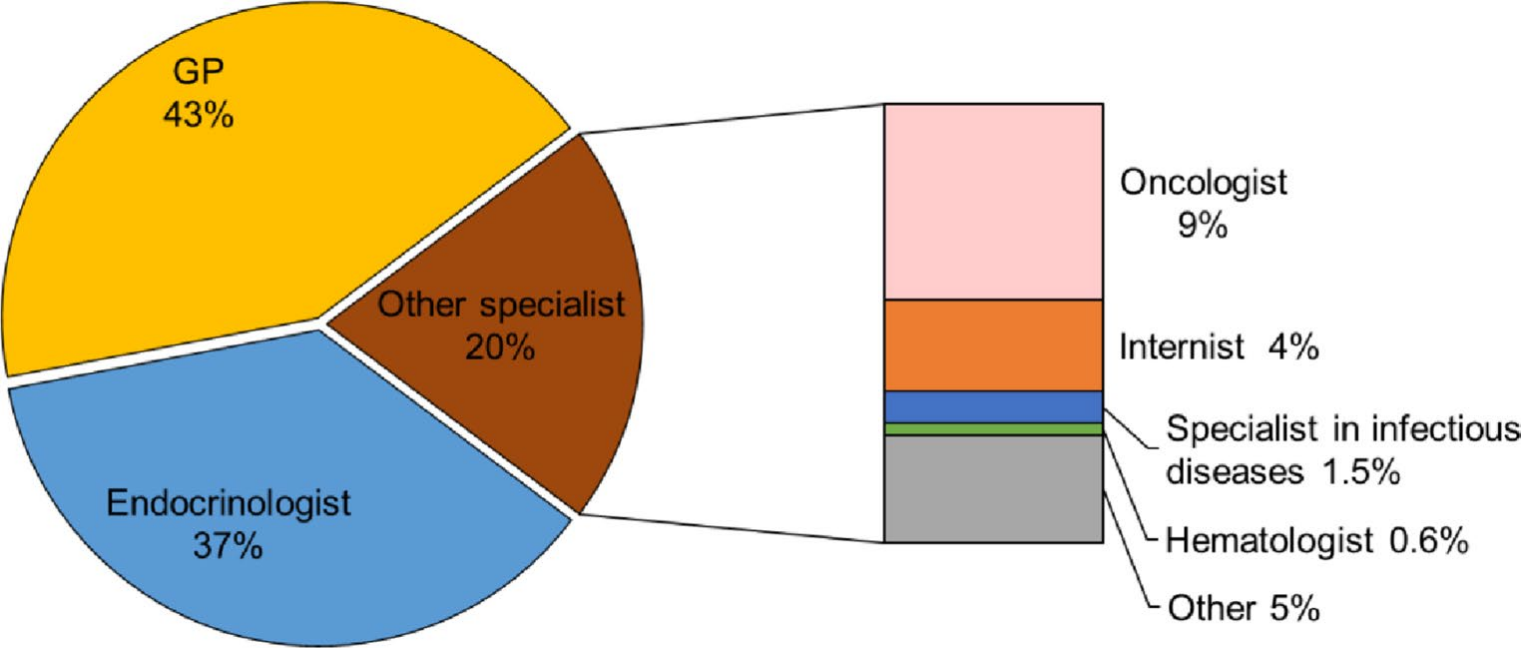
Distribution of the entire cohort according to the source of referral. GP: General Practitioner

Overall, 197 patients (36.8%) were already being followed by our Endocrinology Unit for endocrine/andrological disorders. Bone health evaluation was requested by GPs for 229 patients (42.7%) and by other medical specialists for 110 (20.5%) (Fig. 2). Among the 110 referrals from specialists, 49 (45.4%) came from oncologists, 23 (21.3%) from internists, 8 (7.4%) from infectious disease specialists, and 3 (2.8%) from hematologists (Fig. 2). Notably, 25 out of these 110 (23.2%) were referred for bone consultation during hospitalization following a fracture.

## Comorbidities and medications associated with bone loss

A total of 264 patients out of 536 (49.3%) were affected from at least one comorbidity strongly associated with bone loss as detailed in Table 2. Hypogonadism (including also Klinefelter Syndrome), diabetes mellitus, hypopituitarism and hematological disorders accounted for about 83% of all comorbidities recorded. In detail 310 (57.8%) had *more than* 2 comorbidities/risk factors (Table 1).

**Table 2:** Rates of comordities and medications related to bone loss

CLASSIFICATION OF OSTEOPOROSIS	*N*	%
Patients with unknow cause of osteoporosis	178	33.2
Patients with secondary osteoporosis	358	66.8
**COMORBIDITIES**	*N*	%
Hypogonadism (primary and secondary)	66	12.3
Diabetes mellitus	54	10.1
Klinefelter Syndrome	43	8.0
Hypopituitarism	33	6.2
Hematological diseases	21	3.9
Iron Overload Diseases	2	0.4
Primary hyperparathyroidism	15	2.8
Idiopathic hypercalciuria	7	1.3
Acromegaly	5	0.9
Endogenous hypercortisolism	1	0.2
Malabsorption (including celiac disease, IBD, bariatric surgery)	17	3.2
Infectious diseases (HIV)	7	1.3
Skeletal dysplasia	2	0.4
**Patients with comorbidities – Total**	**264**	**49.3**
**MEDICATIONS INDUCING BONE LOSS**	*N*	%
Corticosteroid treatment	84	15.7
Androgen-deprivation therapy	62	11.6
Proton-pump inhibitors	121	22.6
Loop diuretics	27	5.0
SSRI	20	3.8
Immunosuppressants	15	2.8
**Patients receiving medications linked to bone loss – Total**	**235**	**43.8**

IBD: bowel inflammatory disease; FRAX: 10-year Fracture Risk Assessment

FRAX score was above 3% for hip fractures in 204/471 patients (43%) and it was above 20% for major fractures in 43/469 (9%) (Table 1) showing an unexpected variance between the two scores.

A total of 235 patients (43.8%) reported the use of at least one drug associated with bone loss (Table 2). Of these, 84 patients (15.7% of the entire cohort) were treated with previous or current corticosteroid treatment for more than 3 months with a median duration of 4.0 years (IQR 1.7–7.3); 62 patients (11.6% of the entire cohort) were receiving androgen-deprivation therapy for prostate cancer (Table 2). Corticosteroid and androgen-deprivation therapy together accounted for about 62% of all treatments linked to osteoporosis development.

*Bone parameters at DXA*DXA had already been performed for 427 out of 536 patients (79.7%): DXA at lumbar spine was available for 391 patients (72.9%), whereas DXA at hip site for 413 patients (77.0%).

**Fig. 3:**
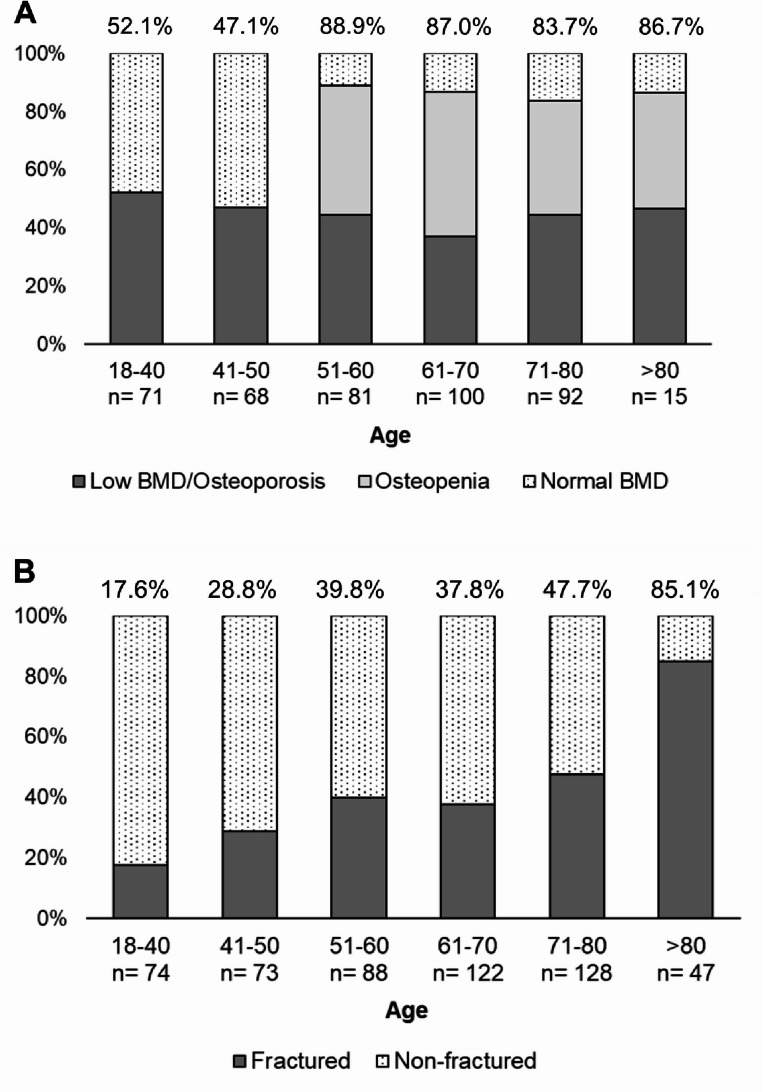
Prevalence of reduced BMD (A) and fractures (B) by decades of age; prevalence is reported as a percentage (%) for each column. Reduced BMD is defined as low BMD for age for decades < 50 years and as osteopenia/osteoperosis for decades > 50 years. BMD: Bone mineral density

According to Z- and T-scores at the lumbar site (L1-L4), patients were classified as follows: 122 (31.2%) had normal BMD, 110 (28.1%) had osteopenia, 102 (26.1%) had osteoporosis, and 57 (14.6%) had low BMD for age. According to Z- and T-scores at the hip site (femoral neck), patients were classified as follows: 156 (37.8%) had normal BMD, 176 (42.6%) had osteopenia, 61 (14.8%) had osteoporosis, and 20 (4.8%) had low BMD for age. Overall, combining lumbar and hip parameters and focusing on patients younger than 50, low BMD for age was found in 69 out of 142 (48.6%) and normal BMD in 73 (51.4%) (Fig. 3A). Considering patients older than 50, 128 patients out of 286 (44.8%) had osteopenia, 121 (42.3%) had osteoporosis, and 37 (12.9%) had normal BMD (Fig. 3A).

## Pharmacological management of bone status before bone specialist’s evaluation

A total of 181 out of 536 patients (33.8%) had received neither calcium and/or vitamin D supplementation nor bone active therapy (Table 1); 219 patients (40.8%) had already received calcium and/or vitamin D supplementation but no drugs; and the remnant 136 patients (44.0%) had received any prior or ongoing bone active therapy.

We focused on the subgroup of 84 men receiving steroid treatment. Of these, 16 (19%) men had no calcium and/or vitamin D supplementation nor ongoing bone active therapy; 44 (52.4%) were receiving calcium and/or vitamin D supplementation; 24 (28.6%) had an ongoing bone active therapy (10 with alendronate, 12 with other bisphosphonates, 1 with teriparatide, and 1 with denosumab).

The same descriptive analysis was repeated for the subgroup of 62 men receiving androgen-deprivation therapy. Of these, 13 (21%) men had no calcium and/or vitamin D supplementation nor ongoing bone active therapy; 40 (63.5%) were receiving calcium and/or vitamin D supplementation; 9 (14.6%) had an ongoing bone active therapy (3 with alendronate, 4 with other bisphosphonates, and 2 with denosumab).

### Rate of osteoporosis-related fractures and comparison between men with and without fractures

**Fig. 4:**
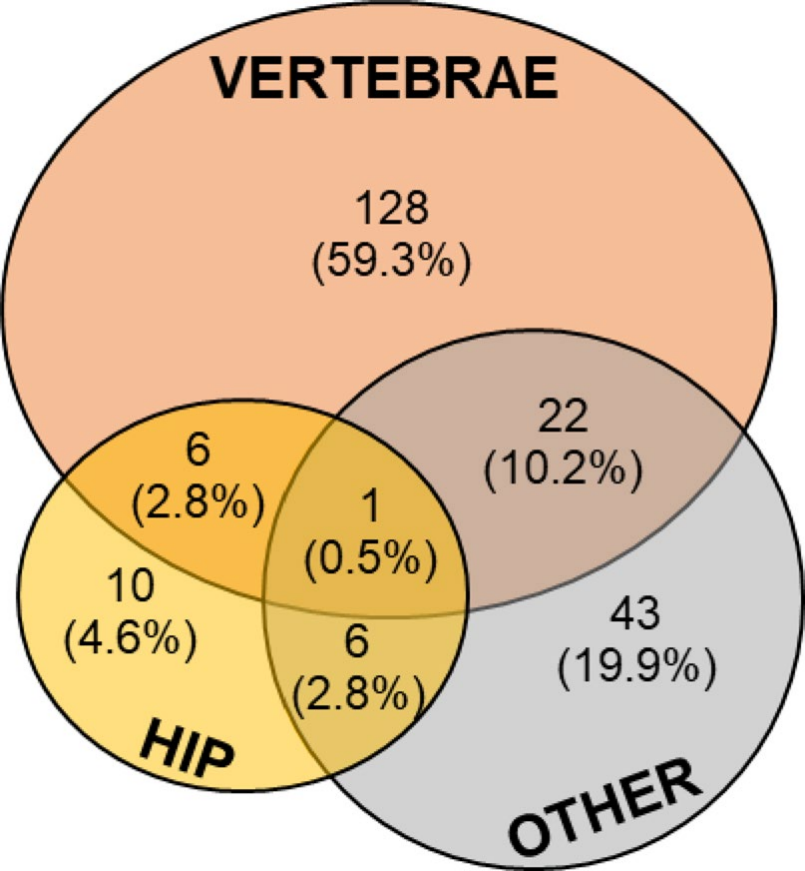
Site of osteoporosis-related fractures documented by radiological examinations

At least one fracture occurred in 216 patients (40.8%) before the bone specialist’s evaluation (Table 3). Vertebral fractures were the most frequent site of fracture occurring in 157 patients (72.7% of fractured patients), alone in 128 patients (59.3% of fractured patients) or in combination with other sites in 29 patients (13.4% of fractured patients) (Fig. 4). Hip fractures occurred in 23 patients (7.3% of fractured patients), alone in 10 patients or in combination with other sites in 13 patients (Fig. 4). Within the thoraco-lumbar spine, L1, L2 and T12 were the most affected being fractured in 58 (26.9%), 41 (19.0%), and 50 patients (23.1%), respectively.

**Table 3:** Comparison of clinical characteristics between fractured and non-fractured patients. Significant dofferences are in bold. Continuous data are reported as median (IQR)

	Fractured patients	Non-fractured patients	*p*-value
*N*	216	320	
**Anthropometric characteristics**			
Age (yrs)	68.5 (55.3–78.0)	59.0 (44.1–69.7)	**< 0.001**
*18–40*	13 (6.0%)	59 (18.8%)	**< 0.001**
*41–50*	21 (9.7%)	52 (16.6%)	
*51–60*	35 (16.2%)	53 (16.9%)	
*61–70*	46 (21.3%)	76 (24.2%)	
*71–80*	61 (28.2%)	67 (21.3%)	
*> 80*	40 (18.5%)	7 (2.2%)	
Height (m)	1.72 (1.68–1.76)	1.75 (1.70–1.80)	**0.001**
Weight (kg)	75 (68–80)	78 (70–88)	**< 0.001**
BMI (kg/m^2^)	24.9 (23.0–27.3.0.3)	25.7 (23.5–28.7)	**0.005**
**Source of referral**			
Endocrinologist	34 (15.4%)	164 (51.4%)	**< 0.001**
General practitioner	142 (65.9%)	87 (27.2%)	
Other specialists	40 (18.7%)	69 (21.4%)	
**Risk factors**,** comorbidities and therapies associated with osteoporosis**			
Age > 65 years	123 (56.9%)	119 (37.9%)	**< 0.001**
BMI < 18 kg/m^2^	3 (1.4%)	4 (1.3%)	0.919
Familial history of osteoporosis	69 (31.9%)	65 (24.2%)	0.057
Parental history of hip fracture	27 (12.5%)	17 (6.7%)	**0.030**
Current smoking	91 (42.1%)	75 (24.8%)	**< 0.001**
Alcohol consumption > 3 units/daily	19 (8.8%)	8 (2.7%)	**0.002**
Inconstant physical activity	188 (87.4%)	205 (71.9%)	**< 0.001**
Low dietary calcium intake	25 (11.6%)	19 (6.7%)	0.053
Major comorbidities inducing bone loss	45 (25.8%)	183 (58.3%)	**< 0.001**
Use of drugs inducing bone loss	119 (55.3%)	116 (37.4%)	**< 0.001**
Chronic steroid therapy	41 (19.0%)	43 (13.7%)	0.101
Androgen-deprivation therapy	15 (6.9%)	47 (15.0%)	**0.005**
Hypogonadism	16 (7.4%)	50 (15.6%)	**0.004**
Primary hyperparathyroidism	3 (1.4%)	12 (3.8%)	0.097
Diabetes mellitus	23 (10.6%)	31 (9.9%)	0.772
FRAX score ≥ 3% for hip fracture	138 (69.0%)	66% (24.4%)	**< 0.001**
FRAX score ≥ 20% for major fracture	40 (20.0%)	3 (1.1%)	**< 0.001**
Comorbidities < 2	77 (35.6%)	149 (46.6%)	**0.012**
Comorbidities *≥* 2	139 (64.4%)	171 (55.2%)	
**DXA parameters**			
Osteoporosis or low BMD	89 (55.6%)	101 (37.7%)	**< 0.001**
Osteopenia	43 (26.9%)	85 (31.7%)	
Normal BMD	28 (17.5%)	82 (35.6%)	
**Management for bone health before bone specialist’s evaluation**			
No prior calcium and/or vitamin D supplementation nor bone active therapy	50 (23.1%)	125 (39.8%)	**< 0.001**
Prior calcium and/or vitamin D supplementation, no bone active therapy	82 (38.0%)	137 (43.6%)	
Ongoing calcium-vitamin D supplementation, prior bone active therapy	10 (4.6%)	16 (5.1%)	
Ongoing bone active therapy	74 (34.3%)	36 (11.5%)	

BMD: bone mineral density. FRAX: 10-year Fracture Risk Assessment

Men with fractures were older compared to men without fractures (*p* < 0.001); accordingly, the prevalence of patients aged above 65 was higher in those with fractures (*p* < 0.001) (Table 3; Fig. 3B). Furthermore, a slight significant difference was found for height, weight, and BMI, being fractured patients shorter and thinner than non-fractured (Tables 1 and 3S). This difference was not confirmed comparing the prevalence of BMI below 18.

Interestingly, we found significant difference in the source of referral. Hence, bone specialist evaluation was requested by different physicians depending on the presence of fractures: in detail, men with fractures were seeking bone evaluation more frequently sent from the GP (142 out of 216, 65.9%) than from endocrinologist or other specialists (*p* < 0.001) (Table 3). The prevalence of fractures in the subgroup of endocrinological patients and of patients sent from another specialist was 34/216 (15.4%) and 40/216 (18.7%), respectively (Table 3).

Although no difference was observed analyzing the general familial history of osteoporosis, history of parental hip fracture was more present in fractured than non-fractured patients (*p* = 0.030) (Table 3).

Concerning lifestyle risk factors for osteoporosis, smoke (*p* < 0.001), alcohol consumption (*p* = 0.002), and inconstant physical activity (*p* < 0.001) were more frequent in the subgroup with fractures, whereas no difference was found for the calcium dietary intake (Tables 1 and 3S). FRAX score was significantly worse in men with fractures (*p* < 0.001) (Table 3).

The use of medications inducing bone loss was more frequently reported in men with fractures compared to non-fractured (55.3% vs. 33.4%, *p* < 0.001) (Table 3). Of note, androgen-deprivation therapy was more frequent in non-fractured than fractured patients (*p* = 0.005) and even hypogonadism (*p* = 0.004) were more prevalent in non-fractured patients (Table 3).

In the subgroup of fractured patients, the majority of subjects had more than 2 risk factors/comorbidities inducing osteoporosis (64.4% vs. 35.6%, *p* = 0.012) (Table 3).

For patients with available DXA, the distribution of normal BMD, osteopenia and osteoporosis was significantly different among subgroups (*p* < 0.001) (Table 3). Among the 110 patients with normal BMD, 82 (74.5%) had no history of fractures whilst 28 (25.5%) had a positive history of fractures. In detail, osteoporosis was more prevalent in subjects with fractures (55.6% vs. 37.7%, respectively), as expected. Notably, up to 28/216 (17.5%) of men with fractures presented with normal BMD (Table 3); these patients were younger compared to fractured patients with osteoporosis/osteopenia based on DXA parameters (49.4 [IQR 42.2–73.7] vs. 65.4 [IQR 55.3–73.5], *p* = 0.019). Considering patients without an available DXA, 60 subjects out of 111 (54.1%) came from the fractured and 51 (45.9%) from the non-fractured subgroup.

No difference was found for single risk factors, comorbidities and drugs related to osteoporosis in the comparison between fractured patients with normal BMD and those with reduced BMD, except for alcohol consumption that was more prevalent in the fractured subgroup (21.4% vs. 6.8%, *p* = 0.016). We compared the score obtained from the sum of risk factors and comorbidities between fractured and non-fracture patients and we found that subjects with at least 2 risk factors/comorbidities had 50% greater risk of fracture (*p* = 0.012, OR 1.57, 95%CI 1.11–2.23). In agreement, at ROC curve analysis a score between 1 and 2 identified fractured patients with 64.4% sensitivity and 46.6% specificity.

Finally, difference was found in the management of fractured and non-fractured patients before the bone specialist’s evaluation (*p* < 0.001) (Table 3). The percentage of patients receiving bone active therapy was higher in fractured than non-fractured subgroup (34.3% vs. 11.5%, respectively), whereas the percentage of treatment-naïve patients was higher among non-fractured; the use of calcium and/or vitamin D supplementation was similar comparing the two subgroups (Table 3). However, among fractured patients 50 (23.1%) did not receive either calcium/vitamin D supplementation or bone active therapy, and 82 (30%) received only bone active therapy.

## Discussion

This study provides, for the first time, a real-life overview of the clinical profile of men seeking consultation for bone health at a tertiary, internationally recognized academic referral Centre for the management of male osteoporosis [18, 24]. In this highly selected cohort, nearly half of the patients were diagnosed with a severe form of osteoporosis, reporting at least one documented osteoporosis-related fracture at the time of the first visit. Consistent with the literature, patients’ medical history revealed a high prevalence of multiple risk factors and comorbidities strongly associated with bone loss [31–33]. Despite this, most had never received calcium or vitamin D supplementation, nor bone-active therapy prior to be addressed to a tertiary center. This study also sheds light on referral pathways, providing valuable insights into the real-life workflow related to this still underrecognized disease.

One of the most striking findings is the high prevalence of osteoporosis-related fractures at the initial bone specialist evaluation: 41% of the overall cohort had at least one fracture, with 13.5% presenting with multiple fractures. As expected, fractures rates were highest among elder men (84–89%) but also significantly present in those under 50 years (18–38%) of age. Vertebral fractures were the most frequent (72.7%), particularly at L1 and T12 [34, 35], followed by L2 and T8 [35]. These data support previous observations that the occurrence of an osteoporotic-related fracture leads to bone specialist referral in only a minority of cases—around 4%—and that approximately 40% of men diagnosed with osteoporosis have already sustained one or more fractures by the time of their first visit [15, 31, 36]. Alarmingly, underdiagnosis also persists among hospitalized patients [37]. The relevant percentage of fractures observed in men under 50 represents a novel and important finding, as most existing data focus on older populations [15, 31, 36]. This indicates that men often come to medical attention too late—after fractures have occurred—highlighting a missed opportunity for early intervention to reduce the risk of re-fracturing.

The situation is further worsened by the low rates of calcium/vitamin D supplementation and bone-active therapy in men with fractures and/or low BMD [31, 38]. One in five patients with fractures had never received any form of bone-specific treatment or supplementation. Likewise, only a small proportion of patients undergoing therapies known to promote bone loss—such as corticosteroids or androgen deprivation—were receiving appropriate preventive bone care. This state of affairs needs to be considered in light of the Italian Health Service organization for the management of osteoporosis and of the local implementation in the province of Modena that provides for an initial action of the GP in terms of diagnosis, prevention, and therapy (Fig. 1).

Our findings indicate that patients with fractures were more frequently referred by GP (65.9%) than by specialists (34.1%), suggesting that the initial management pathway often begins in primary care. These data underscore the persistent gap in the recognition and management of osteoporosis in men, particularly in primary healthcare [16, 19, 21], despite this issue having been acknowledged for over two decades [16, 39]. To improve referral pathways, several strategies could be considered. Strengthening communication and collaboration between GP and specialist services may facilitate more timely and appropriate referrals. The development of standardized referral protocols and digital decision-support tools could help ensure that patients are directed to the most suitable level of care. In addition, continuing medical education programs focusing on fracture assessment and referral criteria might enhance practitioners’ confidence and accuracy in identifying cases that require specialist evaluation. Implementing such measures could ultimately optimize patient flow, reduce delays in diagnosis and treatment, and improve overall outcomes for individuals with fractures.

During the first clinical evaluation at our outpatients’ bone clinic, through an accurate medical interview aiming at detecting risk factors/conditions affecting bone, we found that 41% of patients presented with a comorbidity causing osteoporosis, whilst up to 62% was receiving medications inducing bone loss and 54% of fractured patients had never undergone DXA scanning. It is important to emphasize that these data were gathered before any diagnostic testing, based solely on medical history. Therefore, it is reasonable to assume that these percentages would increase if further laboratory or imaging tests were performed, as previously reported [14].

Likewise, laboratory investigations often reveal additional secondary causes of osteoporosis in men, highlighting the critical need for comprehensive diagnostic workups [3, 18, 21]. Yet, most patients had never received such evaluations prior to their visit to a bone specialist in this study. This is further confirmed by FRAX scores, with 43% of patients having a high estimated risk for hip fracture (>3%). While both GPs and endocrinologists generally consider a full osteoporosis workup appropriate in men with high FRAX scores, prior fractures, or chronic corticosteroid or androgen deprivation therapy [40] or presence of diseases causing secondary osteoporosis [41], the proportion of patients actually referred for such assessments remains unacceptably low [38, 42, 43].

DXA findings revealed that the majority of patients over 50 had osteoporosis (42.3%) or osteopenia (44.8%), with only 12.9% showing normal T-scores. Among those under 50, approximately half had abnormal BMD, while the remaining 51.4% had normal values. Clinically relevant is the observation that 17.5% of men with fractures had normal BMD and 26.9% had osteopenia, indicating that DXA alone may not fully capture fracture risk. Hence, bone loss detected by DXA is not the entire problem, but rather part of a syndrome leading to fracture in line with what experts’ opinions emphasize [44]. This supports the concept that bone fragility can exist even in the presence of non-osteoporotic BMD values, particularly in secondary osteoporosis, which is more common in men [15]. The coexistence of osteoporosis-related fractures and normal BMD complicates diagnosis, especially considering the absence of male-specific normative DXA values, which may contribute to false-negative results [18, 21]. These findings emphasize the need for increased awareness of the limitations of DXA and the importance of comprehensive risk assessment. There is a clear need for educational initiatives targeting GPs and specialist physicians, aimed at increasing recognition of male osteoporosis, especially secondary forms. Bone specialists must also take a proactive role in promoting education and research to bridge the persistent knowledge and management gap. In clinical practice, a proper diagnostic work-up—including both laboratory and imaging studies—should be offered to all men at risk for osteoporosis, regardless of age, to uncover potential secondary causes [17, 18, 21]. This approach must extend beyond BMD measurement alone, as secondary osteoporosis and fractures may be present even with normal DXA values.

A major strength of this study is the 14-year observation period and the relatively large sample size, which spans a wide age range, including younger adults. Data on bone health and management were comprehensive, covering key aspects such as comorbidities, fracture history, densitometric measurements, and prior treatments. Many risk factors and lifestyle habits were derived from self-reports and chart reviews, introducing a potential recall and reporting bias. However, unlike many studies relying on electronic health records or ICD coding [14, 15, 45], data in this study were collected directly by clinicians using a uniform clinical approach and centralized documentation system within a structured and dedicated service for male osteoporosis. The main limitations include its retrospective design and potential underestimation of fracture prevalence, as non-clinical fractures may have been missed. Moreover, as the study was conducted in a tertiary care center, the patient population is likely to include individuals with more severe or complex health conditions than those typically managed in primary or community care. This setting may have introduced a selection bias, leading to an overrepresentation of patients with osteoporosis and fragility fractures and, consequently, to higher observed prevalence rates compared with less selected populations. Therefore, caution is warranted when extrapolating these findings to the general population. Nevertheless, the clinic serves a wide geographic area, including referrals from other regions, which enhances the clinical relevance of the results. It also should be noted that the specific organization of the Italian National Service and its structure within the Province of Modena may limit the applicability of these findings to other healthcare settings. Future studies conducted in broader contexts, including primary and community-based care, would be valuable to confirm these observations. Finally, as the study population is predominantly Caucasian (>99%), the findings may not be fully applicable to other racial or ethnic groups.In conclusion, osteoporosis in men presents with a high rate of fragility fractures (about 50%) at the first bone specialist’s consultation in a male cohort referring to a tertiary academic medical Centre. Most had not received appropriate evaluation or treatment beforehand. A comprehensive work-up—including laboratory testing and imaging—should be recommended for all at-risk men, as bone fragility often extends beyond what BMD alone can reveal, especially in case of secondary osteoporosis. These findings underscore the urgent need to raise awareness of male osteoporosis, especially in primary care. Educational initiatives for both physicians and patients are essential, along with greater research efforts to address existing diagnostic and therapeutic gaps.

## Supplementary Information

Below is the link to the electronic supplementary material.

Supplementary Material 1

## Data Availability

The datasets generated during and/or analysed during the current study are available from the corresponding author on reasonable request.
