# Supplementary material for: Too late, too often: missed opportunities in male bone health: a real-world portrait from a 14-year specialist referral experience
Source: J Endocrinol Invest. 2025 Dec 29;49(3):653–64. doi: 10.1007/s40618-025-02753-8 (PMC13017997; doi:10.1007/s40618-025-02753-8)
Supplement: Supplementary file 1 — Supplementary Material 1 [file 40618_2025_2753_MOESM1_ESM.docx]

**Table 1S. Logistic regressions with osteoporotic fracture as outcome.**

***[Footnote to Table 1S]:*** *OR: Odd ratio; CI: Confidence Interval;* *FRAX: 10-year Fracture Risk Assessment; BMD: Bone Mineral Density.*

|  | **OR [95% CI]** | **p-value** |
| --- | --- | --- |
| **Anthropometric characteristics** | | |
| Age (yrs) | 1.042 [1.029-1.055] | **<0.001** |
| Decade of age |  |  |
| *18-40* | - | **-** |
| *41-50* | 1.535 [1.353-1.742] | **0.110** |
| *51-60* | 3.099 [1.485-6.464] | **0.003** |
| *61-70* | 2.840 [1.408-5.730] | **0.004** |
| *71-80* | 4.272 [2.139-8.534] | **<0.001** |
| *>80* | 26.813 [9.848-73.006] | **<0.001** |
| Height (m) | 0.952 [0.927-0.977] | **<0.001** |
| Weight (kg) | 0.971 [0.958-0.984] | **<0.001** |
| BMI (kg/m^2^) | 0.936 [0.897-0.976] | **0.002** |
| **Source of referral** | 1.858 [1.456-2.371] | **<0.001** |
| Endocrinologist | - | **-** |
| General practitioner | 8.143 [5.135-12.913] | **<0.001** |
| Other specialists | 2.888 [1.681-4.960] | **<0.001** |
| **Risk factors, comorbidities and therapies associated with osteoporosis** | | |
| Age >65 years | 2.201 [1.547-3.130] | **<0.001** |
| BMI <18 kg/m^2^ | 0.925 [0.205-4.176] | 0.919 |
| Familial history of osteoporosis | 1.473 [0.988-2.197] | 0.057 |
| Parental history of hip fracture | 2.000 [1.059-3.778] | **0.033** |
| Current smoking | 1.228 [0.764-1.973] | 0.397 |
| Alcohol consumption >3 units/daily | 3.496 [1.501-8.144] | **0.004** |
| Inconstant physical activity | 2.717 [1.683-4.387] | **<0.001** |
| Low dietary calcium intake | 1.842 [0.986-3.441] | 0.055 |
| Use of drugs inducing bone loss | 2.073 [1.455-2.953] | **<0.001** |
| Chronic steroid therapy | 1.509 [0.945-2.409] | 0.085 |
| Hypogonadism | 0.246 [0.145-0.419] | **<0.001** |
| Primary hyperparathyroidism | 0.362 [0.101-1.296] | 0.118 |
| Diabetes mellitus | 1.111 [0.629-1.963] | 0.717 |
| FRAX score ≥3% for hip fracture | 6.913 [4.596-10.400] | **<0.001** |
| FRAX score ≥20% for major fracture | 22.167 [6.747-72.829] | **<0.001** |
| Comorbidities > 2 | 1.573 [1.103-2.243] | **0.012** |
| **DXA parameters** | | |
| Osteoporosis or low BMD | 2.581 [1.542-4.319] | 101 (37.7%) |
| Osteopenia | 1.482 [0.843-2.605] | 0.172 |
